# *JAK2* Variant and Parkinsonian Syndromes: Coincidence or Pathophysiological Link?

**DOI:** 10.1002/mdc3.70815

**Published:** 2026-09-08

**Authors:** Elena Ardila Jurado, Koustubh Bavdhankar, Divyani Garg, Francesca Magrinelli, Huw R. Morris, Kailash P. Bhatia

**Affiliations:** ^1^ Department of Clinical and Movement Neurosciences, UCL Queen Square Institute of Neurology University College London London United Kingdom; ^2^ Department of Neurology All India Institute of Medical Sciences New Delhi India

**Keywords:** JAK2, myeloproliferative neoplasms, neuroinflammation, parkinsonism, Parkinson's disease

## Abstract

**Background:**

*JAK2* variants are a hallmark of myeloproliferative neoplasms (MPNs), including polycythemia vera and essential thrombocythemia. These disorders are often associated with thrombotic and inflammatory complications. From a movement disorder perspective, chorea is a rare but well‐recognized neurological occurrence in this context, whereas parkinsonism has received limited attention.

**Objectives:**

To describe parkinsonian phenotypes in patients with *JAK2*‐mutated MPNs and explore possible pathophysiological links.

**Methods:**

We identified five patients with a *JAK2*‐mutated MPNs and parkinsonism and reviewed their demographic and clinical features, neuroimaging, and levodopa response.

**Results:**

Parkinsonian phenotypes were heterogeneous, including Parkinson's disease (*n* = 2), atypical parkinsonism (*n* = 1), motor neuron disease with parkinsonism (*n* = 1), and chorea followed by parkinsonism (*n* = 1). The latter patient developed parkinsonism approximately 22 months after onset of generalized chorea. When available (*n* = 2), DaTscan was abnormal. Levodopa responsiveness was variable.

**Conclusion:**

Although *JAK2*‐mutated MPNs and parkinsonism may coexist coincidentally, recent evidence suggests plausible pathophysiological links, including vascular, inflammatory, immune‐mediated, and treatment‐related mechanisms.

Somatic variants in *JAK2* are associated with Philadelphia chromosome–negative myeloproliferative neoplasms (MPNs), including polycythemia vera (PV), essential thrombocythemia, and primary myelofibrosis (PMF).^1^ Over 95% of patients with PV and around 60% with essential thrombocythemia harbor acquired *JAK2* variants, most commonly the missense variant *JAK2*V617F; exon 12 variants occur in a smaller subset of *JAK2*V617F‐negative cases.^1^, ^2^

JAK2 is a cytoplasmic non‐receptor tyrosine kinase coupled to type I cytokine receptors.^1^ Ligand binding activates JAK/STAT signaling.^3^ STAT proteins, specially STAT3 and STAT5, dimerize and translocate to the nucleus, where they regulate transcription of genes implicated in proliferation, survival, differentiation and inflammatory responses.^1^, ^3^, ^4^ JAK2 also activates non‐STAT pathways, including MAPK and PI3K/AKT, reinforcing myeloid expansion and resistance to apoptosis.^1^, ^4^ Acquisition of a valine‐to‐phenylalanine substitution at codon 617 in the JH2 pseudokinase domain causes disruption of JAK2 autoinhibitory control, resulting in ligand‐independent or hypersensitive signaling and persistent activation of proliferative and inflammatory pathways.^4^, ^5^

Although JAK/STAT signaling has recognized roles in the mature CNS, including neuronal and glial functions, synaptic plasticity, neuroinflammatory responses and neurodegenerative processes,^6^ MPN‐associated *JAK2* variants are acquired mutations mainly detected in hematopoietic lineages; reports in endothelial cells exist, but evidence for their presence in neuronal or glial CNS lineages is lacking.^7^

MPNs are increasingly recognized as inflammatory disorders. *JAK2*‐mutant clones interact with the bone marrow niche and inflammatory cytokine milieu to promote systemic inflammation.^1^, ^5^ Arranz et al reported that mutant Hematopoietic Stem Cell‐driven IL‐1β can disrupt sympathetic neural regulation of the marrow niche in experimental models.^5^

Essential thrombocythemia, and particularly PV, are associated with increased thrombotic risk,^2^ with clinical manifestations encompassing pruritus, erythromelalgia, visual disturbances, splenomegaly and hemorrhagic complications.^2^, ^8^, ^9^ Arterial and venous thromboses, including stroke, myocardial infarction, deep venous thrombosis, pulmonary embolism, and thrombosis at unusual sites (eg, splanchnic veins, cerebral venous sinuses) are major drivers of morbidity and mortality and may occur before MPNs diagnosis.^2^, ^10^, ^11^, ^12^ Both conditions carry long‐term hematologic risks, including progression to myelofibrosis, the risk being higher in PV, and acute myeloid leukemia in a subset of patients (~4%).^2^ Neurological manifestations, such as headache, dizziness, visual symptoms, paresthesia, and tinnitus, are common in PV and essential thrombocythemia, may fluctuate with blood count parameters and improve after hematologic treatment.^9^, ^10^

Amongst movement disorders, chorea is the best‐described phenotype associated with *JAK2*‐mutated MPNs.^8^, ^10^, ^13^, ^14^, ^15^ Although rare (~0.5–5%), it's classically described in older adults and is four times more frequent in women.^14^, ^15^ It may be generalized but more prominent in oromandibular regions and upper limbs,^8^, ^13^, ^15^, ^16^ frequently accompanied by muscular hypotonia and hyporeflexia.^13^, ^14^, ^15^ Symptoms may fluctuate, and improvement after phlebotomy or normalization of blood count has been repeatedly reported.^8^, ^13^, ^15^ While more commonly described in PV, chorea has also been reported in *JAK2*‐positive essential thrombocythemia.^17^, ^18^, ^19^

Despite the well‐established neurovascular burden and recognized MPN‐related chorea, the relationship between *JAK2*‐positive MPNs and parkinsonism has received little attention. Reports are limited and include cases in which alternative etiologies were suspected.^10^, ^20^, ^21^ At the same time, emerging literature has revived interest in a possible link between clonal hematopoiesis and parkinsonian syndromes.^22^ Here, we report a novel series of patients with *JAK2*‐mutated MPNs who developed parkinsonian syndromes and discuss possible pathophysiological links between these two entities.

## Methods

We reviewed clinical records of patients diagnosed with parkinsonism in the context of *JAK2*‐mutated MPNs followed up in the Movement Disorder clinics at the National Hospital of Neurology and Neurosurgery, London, UK, and the All India Institute of Medical Sciences, New Delhi, India. Demographic data, medical history, neurological and systemic clinical manifestations, neuroimaging findings, and treatment were retrospectively collected.

## Results

We identified five patients (three males) with *JAK2*‐mutated myeloproliferative disease and parkinsonism (Table 1). Mean age at parkinsonism onset was 69.4 years (range: 58–80). Three patients (P1,P2,P4) had asymmetric parkinsonism, one (P3) had more symmetric parkinsonism, and one (P5) had an axial‐predominant presentation. Rest tremor was present in one patient (P4). Levodopa responsiveness was variable, resulting in clear benefit in two (P2,P4), mild improvement in two (P1,P3) and limited response in one (P5).

**TABLE 1:** Clinical and hematologic characteristics of patients with JAK2‐mutated myeloproliferative disease and parkinsonian syndromes

	P1	P2	P3	P4	P5
Gender	Female	Male	Female	Male	Male
Ethnicity	White European	Ashkenazi Jews	Asian Indian	White European	White European
Chorea	No	No	Mild, generalized	No	No
Chorea treatment	N/A	N/A	Tetrabenazine (12.5 mg twice daily); Clonazepam (0.5 mg once daily)	N/A	N/A
Interval between chorea and parkinsonism	N/A	N/A	22 months	N/A	N/A
Age at parkinsonism onset (years)	73	70	58	80	66
Phenotype	Asymmetric R>L parkinsonism. Gait imbalance. Dystonic posturing right arm. Spasticity. Pseudobulbar dysarthria.	Hypomimia, asymmetric (L>R) parkinsonism.	Later symmetric parkinsonism and blepharospasm	Asymmetric L>R parkinsonism and left rest tremor	Slow vertical saccades. Axial/symmetric parkinsonism with micrography, palilalia, postural instability.
Levodopa response	Mild	Yes	20% improvement in UPDRS‐III	Yes	Limited, worsening of motor symptoms after discontinuation
Non‐motor symptoms	Cognitive decline and emotional lability	Asymptomatic hypotension, urinary urgency. RBD, hyposmia. Mild cognitive impairment, obsessive behaviors and occasional hallucinations	Constipation	Daytime somnolence, RBD	Urinary urgency, slight cognitive impairment
Instrumental investigations	MRI: small vessel disease; EMG: partial denervation in 3 regions in keeping with anterior horn cell disease and sensory neuropathy	MRI: mild generalized atrophy; DaTscan: abnormal	MRI normal	N/A	MRI: normal; DaTscan: abnormal
Hematologic diagnosis	*JAK2*‐positive essential thrombocytemia	*JAK2*V617F‐related essential thrombocythaemia	*JAK2*V617F‐related polycythemia vera	*JAK2*‐positive essential thrombocythaemia	*JAK2*‐positive polycythemia vera and essential thrombocythaemia
Thrombosis history	No	No	DVT right femoral	No	Transient ischemic attack
Hematological treatment	Hydroxycarbamide	Hydroxycarbamide	Phlebotomy; Hydroxycarbamide was started but stopped due to rash/oral ulcers	Hydroxycarbamide	Hydroxycarbamide

Abbreviations: DVT, deep venous thrombosis; EMG, electromyography; JAK2, Janus kinase 2; L, left; MRI, magnetic resonance imaging; P#, patient; R, right; RBD, REM sleep behavior disorder; UPDRS‐III, MDS Unified Parkinson's Disease Rating Scale Part III.

Brain MRI was available in four patients (P1,P2,P3,P5), showing small vessel disease in P1, mild generalized atrophy in P2, and being normal in P3 and P5. There were no imaging features of Parkinson's Plus condition. DaTscan was abnormal in both patients where performed (P2,P5).

The final clinical diagnosis was Parkinson's disease (PD) in two patients (P2,P4), progressive supranuclear palsy (PSP)‐pure akinesia with gait freezing in P5, motor neuron disease with parkinsonism in P1, and chorea followed by parkinsonism in P3. In P3, generalized chorea preceded the onset of parkinsonism by approximately 22 months. Chorea was treated with tetrabenazine and clonazepam before the later emergence of parkinsonian features. However, the parkinsonian features persisted despite tetrabenazine withdrawal.

Hematologically, most patients were diagnosed with essential thrombocythemia, one had PV and one had PV/essential thrombocythemia overlap. Most patients received hydroxycarbamide. One underwent phlebotomy and discontinued hydroxycarbamide due to adverse effects. Thrombotic history was explicitly documented in two cases: P3 had a prior deep vein thrombosis (DVT) of the lower limb and P5 a transient ischemic attack.

## Discussion

We report five individuals who develop parkinsonism in the context of a MPNs. The question raised is whether the coexistence of *JAK2*‐mutated MPNs and parkinsonism occurs coincidentally or could suggest a shared pathophysiology. An age‐related coincidence is plausible: *JAK2*V617F clonal hematopoiesis is uncommon overall (0.2%) but rises from 0.03% under 30 to 1.1% above 81 years,^23^ paralleling the age‐related increase in parkinsonian syndromes.^1^, ^22^, ^24^ However, a purely coincidental explanation may be incomplete. For instance, Huang et al reported a higher prevalence of parkinsonism in *JAK2*‐CHIP (clonal hematopoiesis of indeterminate potential) carriers than in non‐carriers (15.6% vs 6.3%; OR 2.76).^22^ These data do not establish causality but support the plausibility that *JAK2*‐mutant hematopoiesis can correlate with parkinsonian outcomes.^22^

Several mechanisms may connect *JAK2*‐positive MPNs with basal ganglia dysfunction. A first model is vascular. PV and essential thrombocythemia are prototypical thrombotic disorders,^2^ and MPN‐associated chorea has traditionally been linked to hyperviscosity‐related neostriatal dysfunction with impaired cerebral blood flow and venous stasis/hypoperfusion affecting basal ganglia circuits.^8^, ^13^, ^14^, ^25^, ^26^ Huang et al reported ^99^mTc‐TRODAT‐1 SPECT abnormalities that normalized after phlebotomies in parallel with chorea improvement, suggesting potentially reversible presynaptic disturbance.^15^ Extrapolating to parkinsonism, a reasonable hypothesis is that chronic or recurrent microvascular injury, not detected by standard neuroimaging, in vulnerable basal ganglia circuits, lowers the threshold for symptomatic nigrostriatal dysfunction. This is supported by *JAK2*V617F‐specific vascular biology with an exacerbated thromboinflammation and blood–brain barrier injury in cerebral venous sinus thrombosis^27^ and by the hypothesis that platelet activation and cerebral microthromboembolism may link *JAK2*‐mutant clonal hematopoiesis and parkinsonism.^22^ However, chorea has also been described in *JAK2*‐mutated myeloproliferative disease without marked hematologic changes, arguing against a pure hyperviscosity‐driven mechanism and suggesting broader JAK2 biology may contribute to basal ganglia dysfunction.^16^, ^28^

A second hypothesis emphasizes inflammation. *JAK2*V617F‐mutant hematopoiesis is associated with cytokine dysregulation and inflammatory signaling, including elevated IL‐1β, IL‐6, and TNF‐α^1^, ^29^ and mutated JAK2 can induce PD‐L1‐mediated immune escape via STAT3/STAT5 signaling.^29^ In PD, neuroinflammation and immune dysfunction are increasingly recognized contributors to nigrostriatal degeneration: aggregated α‐synuclein activates microglia and promotes inflammatory signaling, with JAK/STAT pathways playing a central role,^3^, ^30^, ^31^ potentially amplifying the toxicity of α‐synuclein.^3^ Qin et al showed that JAK/STAT inhibition suppresses α‐synuclein‐induced microglial and macrophage activation, dampens inflammatory signaling and prevents dopaminergic neurodegeneration in rat models.^30^ Neuroinflammation seems to play a role not only in parkinsonism,^22^ but also in MPN‐associated chorea^16^ and in other neurodegenerative diseases such as Alzheimer's disease (AD).^32^

Furthermore, a reduced catecholaminergic/serotonergic tone and an excessive platelet‐associated dopamine release in the setting of vascular congestion have been proposed.^8^, ^14^, ^25^ Enhanced dopamine receptor sensitivity secondary to relative estrogen deficit following menopause has been suggested, potentially contributing to the higher frequency of PV‐related chorea in females, despite PV being more common in males.^25^ More recent reports raise the possibility that JAK/STAT signaling may contribute directly to basal ganglia vulnerability, supported by work showing JAK/STAT‐mediated proliferation in striatal progenitor cells and neuroprotective effects of JAK2 inhibition in a striatal lesion model.^33^

Notably, one patient in our series manifested with chorea preceding parkinsonism, raising the possibility of evolving basal ganglia vulnerability. Chorea‐parkinsonism overlap or evolution of one into the other is observed across several basal ganglia disorders including Huntington's disease (HD), HD‐like disorders and neuroacanthocytosis.^34^, ^35^, ^36^ However, extending this framework to *JAK2*‐mutated disease requires caution, as we did not identify prior reports clearly describing a longitudinal transition from *JAK2*‐associated chorea to later parkinsonism and the underlying driver remains unclear. Tetrabenazine exposure is an additional confounder, as dopamine depletion can induce or worsen parkinsonian symptoms. Overall, this case is hypothesis‐generating and supports the possibility of a chorea–parkinsonism continuum in selected *JAK2*‐mutated patients.

Treatment‐related parkinsonism deserves consideration. Hydroxycarbamide exposure may modify neuronal vulnerability. Tan et al^37^ reported that hydroxycarbamide promoted PD‐relevant phenotypes in patient‐derived iPSC dopaminergic neurons, including ER stress, impaired neurite outgrowth, reduced p‐AKT signaling, and increased apoptotic markers, although its clinical relevance in MPN‐treated patients remains uncertain.

CAR‐T cell strategies are being explored for MPNs.^38^ Delayed parkinsonism have been reported after B‐cell maturation antigen‐targeted CAR‐T therapy for multiple myeloma.^39^ None of our patients had received CAR‐T cell therapy. Nevertheless, future cases should be assessed carefully for exposure to emerging cellular therapies to avoid attributing treatment‐related parkinsonism to the MPN or its driver mutation.

JAK/STAT modulation is also being explored in other neurodegenerative diseases, including AD and ALS, where pathway dysregulation has been linked to neuroinflammation, glial/immune interactions, and impaired proteostasis/autophagy. JAK inhibitors have therefore been proposed as candidate therapeutics.^3^, ^32^, ^40^

The main contribution of our case series is not to prove that *JAK2*‐mutated MPNs causes parkinsonism, but to generate testable hypotheses. Our study is exploratory and limited by small sample size, retrospective design and inability to infer causality. JAK2 status alone is unlikely to determine neurologic phenotype; allele burden, co‐mutations, inflammatory tone, vascular comorbidities, and treatment exposure may all influence risk and presentation. This observation may prompt others to report similar cases, helping to strengthen the evidence base and clarify whether the association exceeds chance. Given the growing interest in JAK/STAT modulation across neurodegenerative diseases, this question is timely and may have future translational relevance.

## Author Roles

(1) Research project: A. Conception, B. Organization, C. Execution; (2) Manuscript Preparation: A. Writing of the first draft, B. Review and Critique.

E.A.: 1B, 1C, 2A

K.B.: 1B, 1C, 2A

D.G.: 1B, 2B

F.M.: 1B, 2B

H.M.: 1B, 2B

K.B.: 1A, 2B

## Disclosures

**Ethical Compliance Statement:** We confirm that the approval of an institutional review board was not required for this work, as it is based solely on anonymized clinical information and investigation results collected as part of routine diagnostic care. Informed patient consent was not necessary for this work. The study was conducted in accordance with applicable institutional and ethical standards for the use of anonymized clinical information. We confirm that we have read the Journal's position on issues involved in ethical publication and affirm that this work is consistent with those guidelines.

## Financial Disclosures and Conflicts of Interest

Author disclosures are available in the Supporting Information.

## Supporting information


**Data S1.** COI_disclosure.

## Data Availability

Data sharing not applicable to this article as no datasets were generated or analysed during the current study.

## References

[1] Vainchenker W , Kralovics R . Genetic basis and molecular pathophysiology of classical myeloproliferative neoplasms. Blood 2017;129(6):667–679. 28028029 10.1182/blood-2016-10-695940

[2] Tremblay D , Kremyanskaya M , Mascarenhas J , Hoffman R . Diagnosis and treatment of polycythemia Vera: a review. JAMA 2025;333(2):153–160. 39556352 10.1001/jama.2024.20377 PMC11921015

[3] Hu X , Li J , Fu M , Zhao X , Wang W . The JAK/STAT signaling pathway: from bench to clinic. Signal Transduct Target Ther 2021;6(1):402. 34824210 10.1038/s41392-021-00791-1 PMC8617206

[4] James C , Ugo V , Le Couedic JP , Staerk J , Delhommeau F , Lacout C , et al. A unique clonal JAK2 mutation leading to constitutive signalling causes polycythaemia vera. Nature 2005;434(7037):1144–1148. 15793561 10.1038/nature03546

[5] Arranz L , Sanchez‐Aguilera A , Martin‐Perez D , et al. Neuropathy of haematopoietic stem cell niche is essential for myeloproliferative neoplasms. Nature 2014;512(7512):78–81. 25043017 10.1038/nature13383

[6] Nicolas CS , Amici M , Bortolotto ZA , et al. The role of JAK‐STAT signaling within the CNS. JAKSTAT 2013;2(1):e22925. 24058789 10.4161/jkst.22925 PMC3670265

[7] Farina M , Russo D , Hoffman R . The possible role of mutated endothelial cells in myeloproliferative neoplasms. Haematologica 2021;106(11):2813–2823. 34320782 10.3324/haematol.2021.278499 PMC8561279

[8] Marvi MM , Lew MF . Polycythemia and chorea. Handb Clin Neurol 2011;100:271–276. 21496586 10.1016/B978-0-444-52014-2.00019-7

[9] Billot S , Kouroupi EG , Le Guilloux J , Cassinat B , Jardin C , Laperche T , et al. Neurological disorders in essential thrombocythemia. Haematologica 2011;96(12):1866–1869. 21933860 10.3324/haematol.2011.050005 PMC3232271

[10] Silverstein A , Gilbert H , Wasserman LR . Neurologic complications of polycythemia. Ann Intern Med 1962;57:909–916. 13992952 10.7326/0003-4819-57-6-909

[11] Cerquozzi S , Barraco D , Lasho T , et al. Risk factors for arterial versus venous thrombosis in polycythemia vera: a single center experience in 587 patients. Blood Cancer J 2017;7(12):662. 29282357 10.1038/s41408-017-0035-6 PMC5802551

[12] Smalberg JH , Arends LR , Valla DC , Kiladjian JJ , Janssen HL , Leebeek FW . Myeloproliferative neoplasms in Budd‐Chiari syndrome and portal vein thrombosis: a meta‐analysis. Blood 2012;120(25):4921–4928. 23043069 10.1182/blood-2011-09-376517

[13] Nazabal ER , Lopez JM , Perez PA , Del Corral PR . Chorea disclosing deterioration of polycythaemia vera. Postgrad Med J 2000;76(900):658–659. 11009585 10.1136/pmj.76.900.658 PMC1741765

[14] Bruyn GW , Padberg G . Chorea and polycythaemia. Eur Neurol 1984;23(1):26–33. 6370700 10.1159/000115674

[15] Huang HC , Wu YC , Shih LY , Lo WC , Tsai CH , Shyu WC . Reversible abnormal functional neuroimaging presentations in polycythemia vera with chorea. J Neurol 2011;258(11):2054–2057. 21559940 10.1007/s00415-011-6069-y

[16] Bette S , Moore H . Generalized chorea and JAK2V617F mutation‐positive myeloproliferative disorders. Mov Disord Clin Pract. 2020;7(4):462–463. 32373666 10.1002/mdc3.12935 PMC7197301

[17] Koya Kutty S , Di Lazzaro G , Magrinelli F , Mulroy E , Latorre A , Bhatia KP . Late‐onset chorea in JAK2‐associated essential thrombocythemia. Mov Disord Clin Pract. 2021;8(1):145–148. 33426172 10.1002/mdc3.13105 PMC7780952

[18] Calculli A , Arceri S , Pisani A . Chorea associated with JAK2(V617F)‐positive essential thrombocythemia. Mov Disord Clin Pract 2023;10(1):154–155. 36698994 10.1002/mdc3.13567 PMC9847269

[19] Venkatesan EP , Ramadoss K , Balakrishnan R , Prakash B . Essential thrombocythemia: rare cause of chorea. Ann Indian Acad Neurol 2014;17(1):106–107. 24753674 10.4103/0972-2327.128569 PMC3992746

[20] Herishanu Y , Rosenberg P . Effect of L‐dopa on polycythemia. J Am Geriatr Soc 1977;25(5):218–219. 853205 10.1111/j.1532-5415.1977.tb00302.x

[21] Pratesi A , Vella A , Pasini E , Salvi F , Mascalchi M . Parkinsonism in polycythaemia vera probably due to manganism. Mov Disord 2008;23(16):2420–2421. 18823038 10.1002/mds.22319

[22] Huang CE , Chen YY , Lu CH , et al. Prevalence and clinical impact of JAK2‐CHIP: association with parkinsonism and hematologic changes in a population cohort. J Formos Med Assoc 2025;S0929‐6646(25):00523‐6. 10.1016/j.jfma.2025.09.039 41058322

[23] Hinds DA , Barnholt KE , Mesa RA , Kiefer AK , Do CB , Eriksson N , et al. Germ line variants predispose to both JAK2 V617F clonal hematopoiesis and myeloproliferative neoplasms. Blood 2016;128(8):1121–1128. 27365426 10.1182/blood-2015-06-652941 PMC5085254

[24] Cordua S , Kjaer L , Skov V , Pallisgaard N , Hasselbalch HC , Ellervik C . Prevalence and phenotypes of JAK2 V617F and calreticulin mutations in a Danish general population. Blood 2019;134(5):469–479. 31217187 10.1182/blood.2019001113

[25] Janavs JL , Aminoff MJ . Dystonia and chorea in acquired systemic disorders. J Neurol Neurosurg Psychiatry 1998;65(4):436–445. 9771763 10.1136/jnnp.65.4.436 PMC2170280

[26] Thomas DJ , du Boulay GH , Marshall J , Pearson TC , Ross Russell RW , Symon L , et al. Cerebral blood‐flow in polycythaemia. Lancet 1977;2(8030):161–163. 69781 10.1016/s0140-6736(77)90179-9

[27] Bourrienne MC , Le Cam Duchez V , Faille D , Farkh C , Solo Nomenjanahary M , Gay J , et al. Exacerbation of thromboinflammation by JAK2V617F mutation worsens the prognosis of cerebral venous sinus thrombosis. Blood Adv 2024;8(12):3330–3343. 38386979 10.1182/bloodadvances.2023011692 PMC11258627

[28] Gribbin C , Yasmin A , Gallagher P . Chorea and polycythaemia vera. Pract Neurol 2024;24(2):134–136. 37891000 10.1136/pn-2023-003739

[29] Prestipino A , Emhardt AJ , Aumann K , O'Sullivan D , Gorantla SP , Duquesne S , et al. Oncogenic JAK2(V617F) causes PD‐L1 expression, mediating immune escape in myeloproliferative neoplasms. Sci Transl Med 2018;10(429):eaam7729. 29467301 10.1126/scitranslmed.aam7729 PMC6034655

[30] Qin H , Buckley JA , Li X , Liu Y , Fox TH 3rd , Meares GP , et al. Inhibition of the JAK/STAT pathway protects against alpha‐synuclein‐induced neuroinflammation and dopaminergic neurodegeneration. J Neurosci 2016;36(18):5144–5159. 27147665 10.1523/JNEUROSCI.4658-15.2016 PMC6123006

[31] Zhang W , Wang T , Pei Z , et al. Aggregated alpha‐synuclein activates microglia: a process leading to disease progression in Parkinson's disease. FASEB J 2005;19(6):533–542. 15791003 10.1096/fj.04-2751com

[32] Rusek M , Smith J , El‐Khatib K , Aikins K , Czuczwar SJ , Pluta R . The role of the JAK/STAT signaling pathway in the pathogenesis of Alzheimer's disease: new potential treatment target. Int J Mol Sci 2023;24(1):864. 36614305 10.3390/ijms24010864 PMC9821184

[33] Ignarro RS , Vieira AS , Sartori CR , Langone F , Rogerio F , Parada CA . JAK2 inhibition is neuroprotective and reduces astrogliosis after quinolinic acid striatal lesion in adult mice. J Chem Neuroanat 2013;48‐49:14–22. 10.1016/j.jchemneu.2013.02.005 23403094

[34] Hardie RJ , Pullon HW , Harding AE , Owen JS , Pires M , Daniels GL , et al. Neuroacanthocytosis. A clinical, haematological and pathological study of 19 cases. Brain 1991;114(Pt 1A):13–49. 1998879

[35] Reilmann R. Parkinsonism in Huntington's disease. Int Rev Neurobiol 2019;149:299–306, Elsevier. 31779817 10.1016/bs.irn.2019.10.006

[36] Cardoso F , Seppi K , Mair KJ , Wenning GK , Poewe W . Seminar on choreas. Lancet Neurol 2006;5(7):589–602. 16781989 10.1016/S1474-4422(06)70494-X

[37] Tan Y , Ke M , Huang Z , et al. Hydroxyurea facilitates manifestation of disease relevant phenotypes in patients‐derived IPSCs‐based modeling of late‐onset Parkinson's disease. Aging Dis 2019;10(5):1037–1048. 31595201 10.14336/AD.2018.1216 PMC6764725

[38] Kong L , Fu C , Song L , et al. Prospects of chimeric antigen receptor T‐cell therapy in myelofibrosis: from immunopathogenesis to therapeutic strategies. Cancers (Basel) 2026;18(9):1493. 42122287 10.3390/cancers18091493 PMC13162667

[39] Van Oekelen O , Aleman A , Upadhyaya B , Schnakenberg S , Madduri D , Gavane S , et al. Neurocognitive and hypokinetic movement disorder with features of parkinsonism after BCMA‐targeting CAR‐T cell therapy. Nat Med 2021;27(12):2099–2103. 34893771 10.1038/s41591-021-01564-7 PMC8678323

[40] Richardson PJ , Smith DP , de Giorgio A , et al. Janus kinase inhibitors are potential therapeutics for amyotrophic lateral sclerosis. Transl Neurodegener 2023;12(1):47. 37828541 10.1186/s40035-023-00380-y PMC10568794

